# 24-h-Ambulatory Blood Pressure Monitoring in Sub-Saharan Africa: Hypertension Phenotypes and Dipping Patterns in Malawian HIV+ Patients on Antiretroviral Therapy

**DOI:** 10.5334/gh.945

**Published:** 2021-10-13

**Authors:** Philipp Kasper, Angellina Nhlema, Andrew de Forest, Hannock Tweya, Thom Chaweza, Beatrice Matanje Mwagomba, Adam M. Mula, Jane Chiwoko, Florian Neuhann, Sam Phiri, Hans-Michael Steffen

**Affiliations:** 1 Clinic for Gastroenterology and Hepatology, University of Cologne, Faculty of Medicine and University Hospital Cologne, Cologne, DE; 2 Lighthouse Clinic, Lilongwe, MW; 3 Heidelberg Institute for Global Health, University Hospital of Heidelberg, Heidelberg, DE; 4 School of Medicine and Clinical Sciences, Levy Mwanawasa Medical University, Lusaka, ZM; 5 Lighthouse Trust, Lilongwe, MW; 6 Department of Global Health, University of Washington, Seattle, US; 7 Department of Medicine, University of North Carolina School of Medicine, Chapel Hill, NC, US; 8 Department of Public Health, College of Medicine, School of Public Health and Family Medicine, University of Malawi, MW; 9 University Hospital Cologne, Hypertension Center, University of Cologne, Faculty of Medicine and University Hospital Cologne, Cologne, DE

**Keywords:** 24-hour ambulatory blood pressure monitoring, sub-Saharan Africa, HIV, abnormal blood pressure dipping, white-coat hypertension, masked hypertension

## Abstract

**Background:**

Cardiovascular disease and especially hypertension are a growing problem among people living with HIV (PLHIV) on antiretroviral therapy (ART) in sub-Saharan Africa.

**Objectives:**

As robust data on hypertension phenotypes associated with distinct cardiovascular risks among PLHIV are limited, we aimed to assess the frequency of white-coat (WCH), masked (MH) hypertension, and blood pressure dipping-patterns in a group of Malawian PLHIV.

**Methods:**

As part of the prospective Lighthouse-Tenofovir-Cohort-Study, we analyzed clinical, laboratory and 24-h-ambulatory blood pressure monitoring (ABPM) data of PLHIV from urban Lilongwe with treated or untreated hypertension or raised office blood pressure (OBP) during routine study-visits.

**Results:**

118 PLHIV were included and data of 117 participants could be analyzed. Twenty–four-hour ABPM normotension was found in a total of 73 PLHIV including 14/37 on antihypertensive treatment (37.8%). Using strict definitions, i.e. normal OBP plus normal mean BP for all periods of ABPM, controlled hypertension was found in only 4/37 (10.8%) PLHIV on antihypertensive treatment while true normotension was observed in 10/24 untreated patients (41.7%) with previously diagnosed hypertension and 22/56 patients (39.3%) without a medical history of hypertension. WCH with normal BP during all periods of 24-h-ABPM was identified in 12/64 OBP-hypertensive PLHIV (18.8%), primarily in patients with grade 1 hypertension (11/41 patients; 26.8%). MH was found in 17/53 PLHIV with OBP-normotension (32.1%), predominantly in patients with high normal BP (11/20 patients; 55%). The estimated glomerular filtration rate tended to be lower in MH compared to strictly defined normotensive PLHIV (92.0±20.4 vs. 104.8±15.7 ml/min/m²). 64.1 percent of PLHIV (59.5% with 24-h hypertension and 66.7% with 24-h normotension) had abnormal systolic dipping.

**Conclusion:**

The high prevalence of WCH and MH with signs of early renal end-organ damage and an abnormal dipping in approximately 2/3 of PLHIV warrants further investigation as these factors may contribute to the increased cardiovascular risk in PLHIV in resource-limited settings like Malawi.

**Clinical Trial Registration:**

https://clinicaltrials.gov (NCT02381275), registered March 6th, 2015.

## Introduction

The increase in survival of people living with HIV (PLHIV) is accompanied by a rising number of patients suffering from non-communicable diseases (NCD) [1,2,3]. In particular cardiometabolic diseases and HIV are increasingly intertwined in sub-Saharan Africa (SSA), and 20% to 40% of PLHIV from the region, corresponding to 5 to 10 million individuals, are estimated to be hypertensive [4,5]. Heart failure in SSA is most commonly due to hypertensive heart disease [6], and according to a recently published meta-analysis, the prevalence of arterial hypertension rises from 10.5% in untreated HIV patients to 14.5% among PLHIV exposed to antiretroviral therapy (ART) [7]. Hypertension contributes considerably to the NCD burden as hypertensive PLHIV are at a higher risk of cardiovascular events and suffer from increased all-cause mortality compared to hypertensive HIV-uninfected individuals or normotensive PLHIV [8,9].

Twenty-four-hour ambulatory blood pressure monitoring (ABPM) has been established as the most accurate non-invasive method to diagnose arterial hypertension and therefore is recommended in the European and latest US guidelines [10,11]. ABPM can characterize circadian blood pressure (BP) profiles, including nocturnal BP decline (dipping), and has been shown to be superior to office blood pressure (OBP) measurements in predicting total and cardiovascular mortality [12,13,14]. Furthermore, ABPM allows the identification of white-coat hypertension (WCH), i.e. isolated office hypertension with normal out-of-office BP, or masked hypertension (MH), characterized by normal office and elevated out-of-office BP [15]. Although the epidemiological burden of hypertension among PLHIV in SSA is well recognized [16], the availability of ABPM in SSA is limited and data regarding the prevalence of different hypertension phenotypes are scarce. However, this information is very important as each phenotype is associated with different prognostic implications regarding end-organ damage and cardiovascular risk [17,18].

A recently published systematic review revealed a high burden of hypertension in Malawi with prevalence estimates ranging from 15.8 to 32.9% and in particular hypertension control has been declared as one major priority in a current NCD-focused implementation research plan in Malawi [19,20]. As part of the prospective Lighthouse Tenofovir Cohort (LighTen) Study, we performed ABPM to investigate hypertension control rates and phenotypes as well as diurnal BP-patterns in the cardiovascular risk group of PLHIV on ART.

## Methods

### Study population

The prospective LighTen-study (ClinicalTrials.gov NCT02381275), which has been conducted since 2014 at the Lighthouse Clinic in Lilongwe, Malawi, analyses changes in kidney function of PLHIV after initiating a tenofovir-based ART. The study was conducted in accordance with the Declaration of Helsinki and was approved by the Ethical Committees of the involved German universities of Cologne and Heidelberg and the National Health Sciences Research Committee of the Ministry of Health, Malawi. Adult, ART-naïve patients (≥18 years) with confirmed HIV infection were included after they had given written informed consent. Patient demographics, medical history, laboratory values, current medication, and anthropometric data were recorded at study entry as well as during regular study visits. The present study is a subgroup analysis of participants with self-reported hypertension (treated or untreated), newly detected hypertension (BP ≥ 140/90 mmHg on ≥2 measurements at ≥3 occasions) or raised BP during ≥2 routine study visits. At the time when 24-h-ABPM equipment became available at the Lighthouse Clinic in 2017, participants still active and fulfilling these criteria were contacted and asked to participate. 24-hour-ABPM measurements were performed between August 2017 and December 2018. Reimbursement for the cost of the additional trip to and from the clinic and a meal was granted to the study participants. The recruitment had to be stopped because of financial constraints. However, the enrolled study participants were similar to not-enrolled patients with respect to demographic and clinical characteristics (Supplementary Table 1).

**Table 1:** Baseline characteristics of the study population.

PLHIV study population	

Total, n	117

**Demographic and anthropometric measures**	

Gender female, n (%)	60 (51.3)
Age, years	45.6 (38.0–51.4)
Body mass index, kg/m²	26.3 (24.1–30.0)
- Male	25.4 (23.2–28.2)
- Female	28.0 (24.7–31.6)
Type 2 diabetes, n (%)	5 (4.3)
Smoking, n (%)	5 (4.3)
Alcohol consumption, n (%)	25 (21.4)
Creatinine (mg/dL)	0.80 (0.68–0.92)
eGFR (mL/min/1.73m^2^)	102.0 (85.0–112.5)
**HIV-status**	

ART regime (%)	100
Duration on ART, months	26 (19.3–33.5)
Viral suppression, <40 copies/ml, n (%)	113 (96.6)
CD4 count (cells/mm^3^)	392 (241–528)
**Hypertension status**	

History of hypertension, n (%)	61 (52.1)
Previous antihypertensive treatment, n (%)	37 (60.7)
Monotherapy with D or A or C	17/1/1
Comb. A + C	2
Comb. D + A/B/C	4/2/0
Comb. D + A + C	6
Comb. D + B + C	2
Comb. D + A + B + C	2

n, number; eGFR, estimated glomerular filtration rate; D, thiazide diuretic; A, angiotensin converting enzyme inhibitor; B, beta-blocker; C, calcium channel blocker; comb., combination therapy.

### Blood pressure measurements and definitions

Study participants underwent ABPM using a non-invasive oscillometric device (Mobil-O-graph™, IEM GmbH, Stolberg, Germany). This device had been validated and received the highest A/A grade according to the British Hypertension Society protocol [21]. The cuff size was adjusted to the upper-arm circumference. The ABPM-procedure was explained to both patients and controls by a trained clinic employee. All participants were instructed to mark the time of going to bed and getting up in the morning as well as symptoms, physical activity or food intake by pressing pre-specified buttons on the ABPM-monitor. In addition, all events should be documented in a logbook with correspondent pictograms. All instructions were given in written form in *English* and the local language *Chichewa* (Supplementary material).

Daytime (BP-measurements every 15 minutes) and nighttime (BP-measurements every 30 minutes) were originally programmed from 6:00 a.m. to 10:00 p.m. and from 10:00 p.m. to 6:00 a.m., respectively. Already during the feasibility phase of the study only a few individuals marked the time of going to bed and getting up in the morning [22]. Hence, explanations on how to use the monitor buttons were intensified. Since the majority of study participants still forgot to use the designated button, daytime and nighttime were manually adjusted (8:00 a.m. to 8:00 p.m. and 0:00 a.m. to 5:00 a.m., respectively). These time-intervals were based on the lowest or highest values, respectively, from available markings done by patients during the recordings. While the transition phases of the diurnal BP variation were thus left out of consideration, the activity or inactivity periods of the patients can be considered realistic. At least 70% of expected measurements were required for further analysis of the data with respect to hypertension phenotypes [15].

OBP was determined as the mean of the following measurements: 1) oscillometric BP measurement (Rossmax CF115f and Omron M300) as part of anthropometric measurements after entering the clinic, 2) additional measurement by a qualified nurse if BP >140/90 mmHg and 3) manually induced measurement after positioning of the cuff for the ABPM to the non-dominant upper arm. All measurements were done in sitting position after 5 minutes rest [10,15]. A separate room for unattended BP measurements was not available. According to the current European Society of Cardiology/European Society of Hypertension treatment guidelines the following ABPM thresholds were used to define hypertension [10,15]: mean 24-h BP ≥130/80 mmHg or mean daytime-BP ≥135/85 mmHg or mean nighttime-BP ≥120/70 mmHg. Participants with antihypertensive treatment and ABPM-recordings at or above these thresholds were classified as ‘uncontrolled.’

True normotension as well as controlled hypertension were defined as mean OBP <140/90 mmHg plus mean values below all ABPM thresholds, while sustained hypertension was defined as mean OBP ≥140/90 mmHg plus mean 24-h BP ≥130/80 mmHg.

MH was defined as normal OBP with hypertension according to one of the aforementioned ABPM thresholds and WCH was defined as OBP ≥140/90 mmHg with mean values below all ABPM thresholds [15]. Patients on antihypertensive treatment fulfilling these criteria were labelled as masked uncontrolled hypertension (MUCH) and white-coat uncontrolled hypertension (WUCH), respectively.

Since the nocturnal fall in BP is known to be impaired in diabetes [23], the dipping-status was explored only in data sets of non-diabetic PLHIV with at least 20/7 valid diurnal/nocturnal measurements. The prognostic value of nocturnal dipping is related primarily to the systolic BP [24,25]. Therefore, traditional definitions and the continuous night-to-day ratio were applied only to mean systolic nighttime/daytime BP as follows: normal (≥10% but <20%; 0.8 < ratio ≤ 0.9), reduced (1–10%; 0.9 < ratio ≤ 1.0), extreme (>20%; ratio ≤0.8) or reverse (<1%; ratio >1.0) [15].

### Laboratory analysis

Laboratory data were taken from the electronic LighTen-study database including CD-4 cell counts (Pima™ CD4-test, Abbott, Cape Town, South Africa), HIV-RNA plasma levels (m-Pima™ HIV1/2 viral load-test, Abbott, Cape Town, South Africa), and creatinine plasma levels (Erba XL200, Erba, Mannheim, Germany). The estimated glomerular filtration rate (eGFR) was calculated according to the CKD-EPI equation [26].

### Statistical analysis

Descriptive statistics were undertaken. They included the median with interquartile range (IQR) for continuous variables and frequencies for categorical variables. Differences between two groups were analyzed using Student’s t-test for continuous variables and Chi²-test for categorical variables. Differences in mean eGFR between the four hypertension phenotypes were analyzed using Welch’s ANOVA. Statistical analyses were performed using SPSS (Statistical Package for the Social Sciences, Version 25, IBM, Chicago, USA). A two-sided *p*-value <0.05 was considered as statistically significant.

## Results

### Characteristics of the study participants

A total of 118 PLHIV were enrolled in the LighTen-ABPM sub-study. One patient had stopped the 24-h recording prematurely due to discomfort. Thus, 117 PLHIV were included for further analysis (self-reported hypertension n = 40; newly detected hypertension: n = 39; raised BP during ≤2 study visits: n = 38; see Supplementary table 1).

The median age of the PLHIV cohort was 45.6 years (IQR: 38.0–51.4) and 51.3% were female. About two-thirds of the PLHIV were classified as overweight (BMI ≥ 25 kg/m², n = 49) or obese (BMI ≥ 30 kg/m², n = 29). At the time of ABPM, all PLHIV received ART, in all but four patients consisting of tenofovir/lamivudine/efavirenz in a fixed drug combination and the median duration on ART was 26.0 (IQR: 19.0-33.5) months. The median CD4-count was 392 (IQR: 241–528) cells/mm^3^ and all but four patients had viral loads below the lower limit of detection. Study cohort characteristics are shown in Table 1.

At the time of ABPM 52.1 % of PLHIV (n = 61) had a prior diagnosis of hypertension, of whom 37 (60.7%) took antihypertensive medications as prescribed. When follow-up of the LighTen Cohort study ended on October 31^st^, 2019, none of the included patients had died.

### Office blood pressure measurements

**Figure 1:**
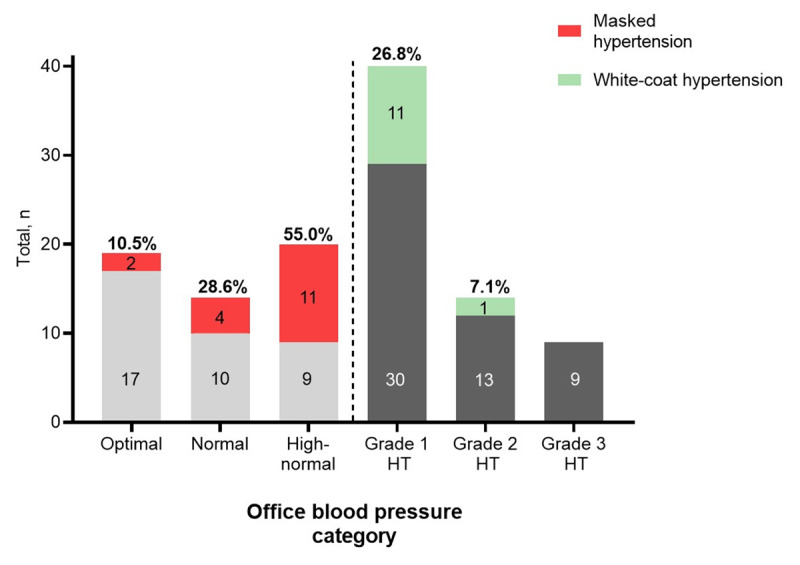
**Office blood pressure categories among PLHIV.** Optimal OBP (<120/80 mmHg; n = 19); normal OBP (120–129/80–84 mmHg; n = 14); high-normal OBP (130–139/85–89 mmHg; n = 20), grade 1 hypertension (140–159/90–99 mmHg; n = 41), grade 2 hypertension (160-179/100-109 mmHg; n = 14), grade 3 hypertension (≥180/110 mmHg; n = 9). Given are absolute numbers within each OBP category and percentages of masked or white-coat hypertension. HT, hypertension.

Elevated OBP was found in 64 of 117 PLHIV (54.7%) (Table 2). Grade 1 office hypertension (in five cases as isolated systolic hypertension) was observed in 41 (64.1%), grade 2 in 14 (21.9%), and grade 3 in nine (14.1%) PLHIV, respectively (Figure 1).

**Table 2:** Office BP and 24-h-ABPM characteristics.

	PLHIV (n = 117)		*p*-value

Office BP status	≥140/90 mmHg (n = 64)	<140/90 mmHg (n = 53)	

Gender (f/m)	34/30	26/27	0.712
Age	46.3 (40.9–53.9)	43.0 (34.5–50.0)	0.096
BMI	27.4 (25.1–31.8)	25.1 (22.9–28.5)	0.057
OBP Mean (mmHg)			
- Systolic	143.3 (133.1–153.0)	124.0 (113.0–129.5)	<0.0005
- Diastolic	96.3 (93.0–102.4)	79.5 (73.8–85.5)	<0.0005
**OBP categories (n)**			

- optimal	–	19	
- normal	–	14	
- high normal	–	20	
- Grade 1 HT	36	–	
- Grade 2 HT	14	–	
- Grade 3 HT	9	–	
- Isolated systolic HT	5	–	
**24-h-ABPM (mmHg)**			

Mean 24-h systolic	122.0 (116.0–131.0)	112.0 (104.5–119.5)	<0.0005
Mean 24-h diastolic	80.0 (76.0–88.8)	71.0 (67.5–76.5)	<0.0005
Mean daytime systolic	124.0 (118.0–134.8)	115.0 (107.0–121.0)	<0.0005
Mean daytime diastolic	84.0 (79.0–91.0)	75.0 (70.5–79.5)	<0.0005
Mean night-time systolic	114.5 (107.0–123.3)	105.0 (99.5–116.0)	<0.0005
Mean night-time diastolic	72.0 (68.0–80.3)	64.0 (59.0–70.0)	<0.0005

BMI, body mass index; OBP, office blood pressure; HT, hypertension; BP, blood pressure; ABPM, ambulatory blood pressure monitoring.

### ABPM characteristics

24-h ABPM normotension was confirmed in only 14 of 37 PLHIV (37.8%) with treated hypertension (Table 3). On the other hand, 59 of 80 PLHIV (73.8%) who were not on antihypertensive medication fulfilled criteria of 24-h normotension, of those, 19 reported to have been previously diagnosed with hypertension based on OBP measurements. However, controlled hypertension, i.e. normal OBP plus normotensive mean BP values during all periods of ABPM, was observed in only 4 of 37 treated patients (10.8%). True normotension according to the same definition was found in 10 of 24 untreated patients (41.7%) with previously diagnosed hypertension based on OBP measurements and 22 of 56 patients (39.3%) with no previously confirmed hypertension diagnosis.

**Table 3:** Hypertension status based on mean 24-h-ambulatory blood pressure data.

Mean 24-h-ambulatory BP	≥130/80 mmHg (n = 44)	<130/80 mmHg (n = 73)	*p*-value

Hypertension status			<0.0005
- treated	23	14	
- untreated	21	59	

BP, blood pressure.

**Figure 2:**
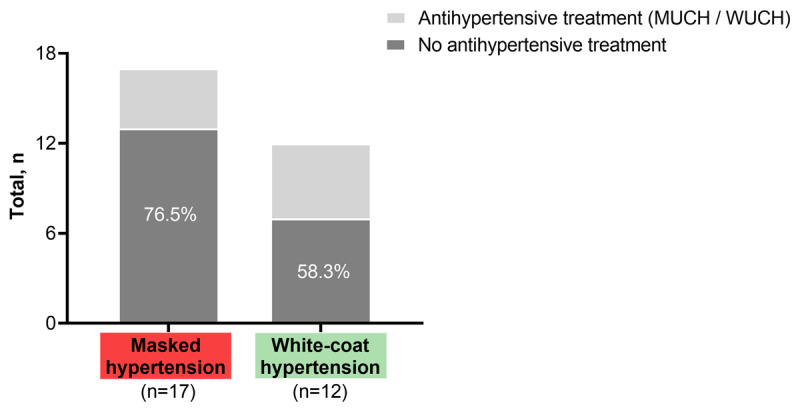
**Hypertension phenotypes and treatment status.** Proportion of patients in each category who did not receive antihypertensive treatment (%). MUCH, masked uncontrolled hypertension; WUCH, white-coat uncontrolled hypertension.

Among PLHIV with elevated OBP, 12 (18.8%, Figures 1 and 2) had WCH of whom seven were untreated (Figure 2). Among patients with OBP grade 1 hypertension (n = 41) WCH was found in 11 (26.8%, Figure 1).

MH was observed in 17 of 53 PLHIV with OBP normotension (32.1%, Figure 2) and 11 of these 17 patients had high normal OBP corresponding to 55% of all patients within this BP category (n = 20, Figure 1). Thirteen of 17 PLHIV (76.5%) with MH received no antihypertensive medication, while four individuals were on antihypertensive treatment (MUCH; Figure 2) and 7/17 (41.2%) suffered from isolated nocturnal hypertension. There was a trend (p = 0.08) for a lower mean eGFR between MH (92.0 ± 20.4 mL/min/1.73m^2^) vs. true normotension/controlled hypertension (104.8 ± 15.7 mL/min/1.73m^2^), WCH (93.3 ± 11.4 mL/min/1.73m^2^), or sustained hypertension (95.3 ± 22.8 mL/min/1.73m^2^). Data sets from 103 PLHIV were eligible to analyze the dipping status (Table 4). An abnormal systolic dipping pattern was found in a total of 66 PLHIV (64.1%) with no significant differences in traditionally defined categories or the continuous night-to-day ratio between those with 24-h ABPM hypertension (59.5%) or 24-h ABPM normotension (66.7%).

**Table 4:** Dipping pattern of PLHIV and 24-h ABPM hypertension status.

	PLHIV (n = 103)		*p*-value

Dipping pattern (total no.)	mABP ≥130/80 mmHg (n = 37)	mABP <130/80 mmHg (n = 66)	

Extreme dipping	2	2	0.317
Normal dipping	15	22	
Reduced dipping	15	38	
Reverse dipping	5	4	
Mean daytime systolic, mmHg	133.0 (127.5–140.5)	114.5 (107.8–119.0)	<0.0005
Mean daytime diastolic, mmHg	89.0 (84.0–97.5)	76.0 (71.8–80.0)	<0.0005
Mean night-time systolic, mmHg	121.0 (115.0–134.5)	105.0 (99.8–112.0)	<0.0005
Mean night-time diastolic, mmHg	74.0 (71.5–88.0)	66.0 (60.8–70.0)	<0.0005
Systolic night-day ratio	0.91 (0.86–0.94)	0.92 (0.89–0.97)	0.527

mABP, mean 24-h ambulatory blood pressure.

## Discussion

While previous studies have shown that hypertensive PLHIV from SSA have an increased risk of adverse cardiovascular events, information on the prevalence of unique hypertension phenotypes with their distinct CVD risk is missing. To the best of our knowledge, this study provides the first comprehensive assessment of MH and WCH among PLHIV in sub-Saharan Africa. The results presented here give a deeper insight into the general difficulties of correctly diagnosing hypertension as cardiovascular risk factor since a relevant number of PLHIV were wrongly labelled as hypertensives needing treatment while the increased cardiovascular risk was unrecognized in quite a number of PLHIV on the basis of OBP measurements alone.

The present study identified MH in 32.1% and 55% of PLHIV with OBP normotension or high-normal BP, respectively. The identification of MH is very important, since patients with MH frequently develop target organ damage prior to transitioning to sustained hypertension and MH confers a cardiovascular risk similar to that of sustained hypertension [27,28,29]. Data on the frequency of MH among PLHIV are scarce and primarily from countries outside SSA (e.g. USA, Argentina) [30,31]. A recent meta-analysis from eight African populations including five from SSA reported a lower MH prevalence of 11% (95% CI: 4.7–19.3) for HIV-uninfected African adults [32]. However, it should be noted that the studies included in this meta-analysis showed a great variation in prevalence estimates, ranging from 5.1 – 41%, used different diagnostic criteria for MH, and had variable methodological quality.

In the present study, MH in PLHIV was accompanied by a reduced eGFR. Recent studies have demonstrated that MH is associated with increased risk for the development of chronic kidney disease [33,34]. Since the presence of renal dysfunction further increases the CVD risk, early detection of PLHIV with MH is essential and needs confirmation in larger study populations.

The high rate of MH among PLHIV might be due to impaired baroreceptor function, autonomic dysfunction and disturbed BP variability, which can be observed in both, subjects with MH as well as PLHIV [8,9,27]. Moreover, psychosocial stressors could contribute to the high MH frequency specifically among this group of Malawian PLHIV, who often live at the subsistence minimum. Hypertension occurs more frequently in individuals with low-income, low-occupational status and elevated psychological stress, all of which can be frequently observed among PLHIV in SSA [35,36,37]. Established risk factors for MH include older age, high-normal systolic and diastolic BP, overweight/obesity, diabetes, smoking, excessive alcohol consumption and African American ethnicity [38,39,40]. HIV-infection might represent another independent risk factor for MH, however, further clarification in larger-scale prospective cohort studies is needed before drawing definite conclusions.

The prevalence of WCH in the present study was 18.8%. In two previous studies from Europe with a relatively small number of cases (43 and 77 PLHIV, respectively), the prevalence of WCH among PLHIV was 39% and 26%, respectively [41,42]. While the frequency of WCH in the present study is slightly lower, it is higher compared to African populations, where a WCH prevalence of 14.8% (95% CI: 9.4–21.1; 8 studies) has been reported in a pooled sample of 4,451 HIV-negative individuals [32]. The higher frequency of WCH among PLHIV in the present cohort compared to data from the general African population might be due to increased levels of anxiety and psychological stress in PHLIV when entering a HIV-clinic.

The clinical management of WCH is debatable. While previous data suggested that patients with untreated WCH (defined by elevated OBP and normotensive daytime ABPM) and normotensive patients were at a similar risk of cardiovascular disease [43], a comprehensive meta-analysis published by Huang et al. found a significant increase in cardiovascular morbidity and total mortality among untreated WCH patients compared to normotensive controls [44]. When an OBP ≥140/90 mmHg plus a normal mean value for the whole 24-h period were applied as the defining criteria, the prevalence of WCH was found to be 4% to 8% in South Africa [45]. Using the ESH criteria [10], the prevalence of WCH in untreated or treated Spanish office hypertensive patients turned out to be 27.2% and 26.1%, respectively, and hypertension-mediated organ damage (e.g. decreased eGFR) was observed at the same rate as in normotensive study participants [46]. In view of these findings from a registry including more than 100,000 patients and due to the lack of clear evidence on whether cardiovascular risk of WCH is reduced by antihypertensive drug treatment, it seems justified to postpone antihypertensive drug treatment in this type of WCH, especially in resource-limited settings, where the availability of antihypertensive drugs is limited, and appropriate resource allocation is needed. Nevertheless, WCH patients require adequate management of concomitant cardiovascular risk factors and should be monitored closely for transition to sustained hypertension.

Besides MH and WCH, an abnormal systolic dipping pattern of BP is also associated with more severe target-organ damage and increased cardiovascular risk [24,25]. In line with preliminary data from the feasibility phase of the present study [22], nocturnal BP dipping was abnormal in the vast majority of patients. In a comprehensive meta-analysis involving seven studies with ABPM data from 420 HIV-infected and 714 HIV-negative individuals, Kent et al. demonstrated that an abnormal diurnal BP pattern was more common among HIV-infected individuals with a pooled odds ratio for non-dipping systolic BP of 2.72 (95% CI: 1.92, 3.85) versus HIV-negative individuals [47]. However, comparability is limited as methods varied with respect to study design, and the majority of subjects were Caucasians. In one of the few studies carried out in SSA, Borkum et al. performed ABPM in black PLHIV from South Africa and observed reduced dipping BP in 65% (43/67) of the study participants [48], which is higher than the 51.5% found here. However, while in their study 61/67 PLHIV were female, the present study included a larger cohort with a more even gender distribution and could confirm that abnormal dipping patterns (64.1%) are a significant problem in Malawian PLHIV.

Uncontrolled hypertension and hypertension unawareness turned out to be further significant problems in the investigated study cohort, both of which are particularly prevalent in rural areas of sub-Saharan Africa [4,49]. A large systematic meta-analysis by Ataklte et al. revealed that less than 40% of hypertensive individuals in the general population of SSA were diagnosed as such, less than 20% of those diagnosed received antihypertensive medication, and BP was controlled, i.e. <140/90 mmHg, in less than 10% of treated individuals [49]. A similar pattern can be observed among hypertensive PLHIV from SSA, where the rate of uncontrolled hypertension is also very high, ranging from 61–85%, and rates of hypertension awareness are typically < 30% [50,51]. In the present study, 37.8% of treated patients had controlled hypertension according to mean 24-h BP values. However, when the strict definition of controlled hypertension was applied, i.e. normal OBP plus normal mean BP values for all periods of ABPM the rate even dropped to 10.8%, much lower than previous findings in hypertensive PLHIV from Malawi, where the BP control rate according to OBP readings was 38% and 30% among patients with hypertension grade 1 or 2, respectively [50]. The reasons for poor hypertension control in SSA are manifold, including irregular supply of antihypertensive drugs with frequent changes in prescriptions (and hence reduced adherence), substandard quality of drugs, shortage of health workers and centers, and poverty in general [52,53]. Another major problem remains the limited availability and low systematic use of out-of-office BP measurement techniques, including ABPM, in HIV-health care. A recent cost-effectiveness analysis from Uganda concluded that the integration of services for NCDs into HIV care would lead to an absolute risk reduction of 1.6% in women and 1.2% in men with estimated net costs between $1,400 to $3,250 per disability-adjusted life year averted [54]. However, estimates relying on models based on OBP-measurements alone to define hypertension may underestimate its true inherent burden among PLHIV in SSA.

Self-reported hypertension represents another important source of bias and seems to be considerably flawed as can be seen in the present study where 10/24 PLHIV with known but untreated hypertension were truly normotensive according to all ABPM thresholds [55].

### Strengths and limitations

This study has several strengths, including the availability of data from a comparably large PLHIV cohort from SSA. In addition, ABPM and OBP were measured following standardized protocols. However, there are also several limitations. Firstly, the absolute number of patients with MH or WCH was overall low, and follow-up was limited with respect to clinical endpoints. More and reliable data on MH and WCH among PLHIV are needed to estimate the true prevalence of hypertension subtypes in this population at increased cardiovascular risk. Secondly, the LighTen study protocol did not include imaging procedures, thus, hypertensive vascular or cardiac damage could not be determined. Thirdly, as ABPM was obtained only on a single occasion and at least for treated patients, the recently shown poor long-term reproducibility of masked or white-coat uncontrolled hypertension has to be taken into account [56]. Fourthly, keeping a diary whilst wearing the ABPM device and using the designated button to indicate the respective monitoring periods has been neglected by a large part of our study participants. Therefore, the evaluation of the periods with daytime physical activity and nocturnal rest needs cautious interpretation. Fifthly, we did not include a group of PLHIV with confirmed OBP normotension during all study visits. While this could have falsely inflated the rate of WCH the opposite holds true for the more important risk factor of MH. However, it probably explains the low total number of patients with true normotension.

## Conclusions

ABPM helped to identify MH with its high cardiovascular risk in a considerable number of PLHIV with normal or high normal BP, while WCH was confirmed in a quarter of patients with grade 1 office hypertension, whose therapy can be postponed in view of a probably favorable cardiovascular prognosis. In addition, a lack of normal nocturnal BP dipping with its inherent unfavorable effects on cardiovascular disease was identified in the majority of PLHIV from urban Malawi.

Implementation of ABPM into HIV care programs in resource-limited settings represents a promising approach to affirm a diagnosis of hypertension and allocate antihypertensive treatment to those who need it the most among in the high-risk population of PLHIV.

## Data Accessibility Statement

Data available on request.

## Additional Files

The additional files for this article can be found as follows:

10.5334/gh.945.s1 Supplementary Table 1. Enrolled vs. not enrolled PLHIV for the LighTen ABPM sub-study.

10.5334/gh.945.s2 Supplementary Material. LighTen-24-h ABPM sub-study event diary and patient instructions.

## Author Information

Philipp Kasper and Angellina Nhlema are shared first authors. Sam Phiri and Hans-Michael Steffen are shared senior authors. Adam M. Mula is deceased.
