# Supplementary material for: 24-h-Ambulatory Blood Pressure Monitoring in Sub-Saharan Africa: Hypertension Phenotypes and Dipping Patterns in Malawian HIV+ Patients on Antiretroviral Therapy
Source: Glob Heart. 2021 Oct 13;16(1):67. doi: 10.5334/gh.945 (PMC8516010; doi:10.5334/gh.945)
Supplement: Supplementary Table 1. — Enrolled vs. not enrolled PLHIV for the LighTen ABPM sub-study. [file gh-16-1-945-s1.pdf]

Supplemental Table 1: Enrolled vs. not enrolled PLHIV for the LighTen ABPM sub-study

	Self-reported hypertension			Newly detected hypertension			Raised BP		
	Enrolled (n=40)	p-value	Not enrolled (n=9)	Enrolled (n=39)	p-value	Not enrolled (n=34)	enrolled (n=38)	p-value	Not enrolled (n=108)
Gender (f/m)	29/11	0.746	7/2	16/23	0.541	16/18	14/24	0.322	51/57
WHO stage 1	17	0.746	2	26	0.979	22	15	0.561	53
WHO stage 2	11		4	4		3	7		22
WHO stage 3	11		3	6		7	15		28
WHO stage 4	1		0	3		2	1		5
age (years)	44.5 (35.3-47.8)	0.126	38.0 (30.5-43.5)	43.0 (38.0-52.0)	0.070	40.0 (32.5-47.0)	41.5 (30.0-49.5)	0.538	39.0 (33.0-44.8)
BMI (kg/m²)	27.7 (23.3-30.5)	0.810	27.5 (23.8-30.6)	27.1 (22.5-30.8)	0.436	24.8 (23.4-27.3)	24.6 (21.7-27.0)	0.307	24.0 (21.0-26.8)
CD4 cell count/µl	314 (110-419)	0.157	391 (237-722)	317 (178-405)	0.609	295 (186-383)	207 (133-413)	0.653	241 (133-407)
eGFR (ml/min/m²)	88.5 (67.3-104.8)	0.468	92.0 (78.0-106.5)	89.0 (74.0-104.0)	0.117	93.5 (83.8-110.8)	88.0 (73.8-105.8)	0.265	96.0 (76.0-109.0)
OBP study entry sys/dia (mmHg)	138.0 (115.3-168.5) / 90.5 (71.0-110.3)	0.500<br>0.675	133.0 (110.0-156.5) / 87 (71.5-99.0)	144.0 (139.0-158.0) / 93.0 (82.0-97.0)	0.821<br>0.912	144 (134.5-152.3) / 93.5 (87.3-98.0)	124.0 (118.5-135.5) / 78.0 (73.0-88.0)	0.851<br>0.466	125.5 (113.0-140.0) / 81.0 (72.0-91.0)
OBP last visit prior to ABPM sys/dia (mmHg)	133.5 (113.0-166.0) / 86.0 (74.3-102.0)	0.423<br>0.366	127.0 (112.5-135.5) / 79 (72.0-88.5)	133.0 (126.0-145.0) / 88.0 (80.0-94.0)	0.153<br>0.872	139.0 (129.5-150.0) / 90.0 (76.0-97.0)	122.0 (110.0-134.5) / 81.0 (72.5-92.5)	0.330<br>0.787	127.0 (116.0-138.0) / 83.0 (75.0-90.0)

WHO, World Health Organization; BMI, body mass index; eGFR, estimated glomerular filtration rate; OBP, office blood pressure; sys, systolic blood pressure; dia, diastolic blood pressure; ABPM, ambulatory blood pressure monitoring. Chi²-test for categorical variabes and Mann Whitney U-test for continuous variables, respectively.
