# Supplementary material for: 24-h-Ambulatory Blood Pressure Monitoring in Sub-Saharan Africa: Hypertension Phenotypes and Dipping Patterns in Malawian HIV+ Patients on Antiretroviral Therapy
Source: Glob Heart. 2021 Oct 13;16(1):67. doi: 10.5334/gh.945 (PMC8516010; doi:10.5334/gh.945)
Supplement: Supplementary Material. — LighTen-24-h ABPM sub-study event diary and patient instructions. [file gh-16-1-945-s2.pdf]

Supplemental Material: LighTen-24-h ABPM Substudy event diary

LighTen-24h-ABPM Substudy Diary

Patient ID: First Name: Last Name:

A. Please **press the arrow sign button** and tick against the relevant picture during the following events in order of occurrence (*Chonde dinani batani la kalozera panthawi yomwe izi zikuchitika ndipo muchonge mmunsimu mwa mndandanda mmene zachitikira):*

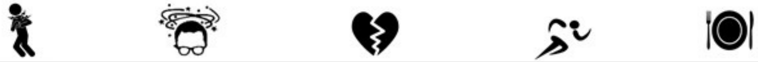

Shortness of breath	Dizziness	Heartache	Physical activity	Meal
Kubanika	*Chizungulire*	*Kumva mtima kupweteka*	*Masewera/majowajowa*	*Kudya*

1.
2.
3.
4.
5.
6.
7.

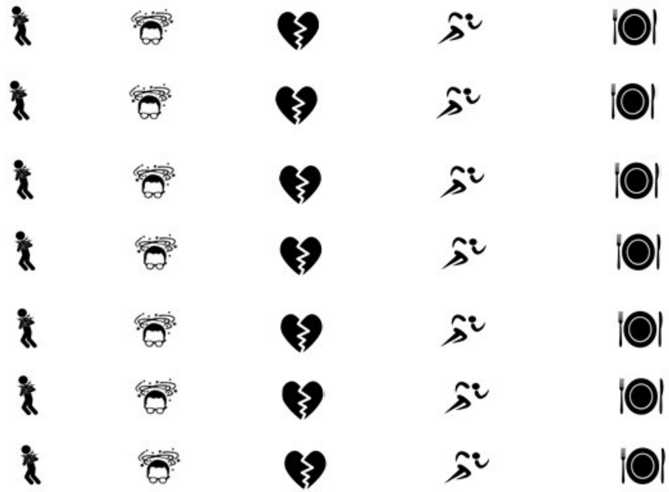

B. Please **press the day/night sign button** once when going to sleep at night and once when waking up in the morning ***(Chonde dinani batani lowonetsa usiku ndi usana kamodzi pomwe mukukagona ndi kamodzi mukadzuka mmawa)***

C. Please **press the start key sign button** after taking medication. Triggers an additional measurement. ***(chonde dinani batani ili mukatha kumwa mankhwala. Mukatero makina ayambanso kuyesa)***

Important instructions for the patient

- The beep signals a measurement. Keep still during the measurement, until the process is complete. Let your arm hang loosely or, when sitting, let your lower arm rest on the table or on a support. Sit with feet flat on the floor, legs not crossed, back supported. Avoid moving your hand during the measurement. Relax and do not talk during the measurement.
- The ON/OFF key permits the device to be switched on and off if a measurement is not required at the set time. To switch off, press the key for longer than 2 seconds.
- A measurement can be interrupted at any time by pressing any of the keys. This deflates the cuff.

Warnings:

- The cuff may only be worn on the upper arm and in every case you should ensure that neither the shoulder strap nor the air tube can ever become wrapped around your neck. The air tube should therefore always be worn under the outer clothing (even at night).
- Take care to ensure that the tube of the cuff cannot be kinked or compressed, particularly while sleeping.
- If the measurement causes you pain, switch off the device, take off the cuff and inform your doctor.
- The blood pressure monitor must not come into contact with liquids. Do not wear the equipment in the shower! If, for example, water has entered the device, it may no longer be used. Switch off the device and take out the batteries.
- Serious malfunctions are indicated by a continuous beep. If a continuous beep sounds, switch off the device, take off the cuff and inform your doctor.
- The cuff connection (air connector) must always engage with an audible click. Otherwise there will be a leaky connection between the Mobil-O-Graph 24h PWA® and the tube that will produce incorrect measurements.

In case of urgent questions you can contact one of the following numbers:

- .
-
-

Malangizo ofunika

- Makina akalira zikusonyeza kuti ayamba kuyesa. Panthawi imeneyi musasunthe komanso musayankhule mpaka amalize kuyesa. Ngati mwakhala pansi, tsamiritsani nkono omwe uli ndi makina patebulo kapena pa miyendo yanu. Ngati mwakhala pa mpando musasanjikize miyendo koma ngati mwakhala pansi mukhale motambalala, osasanjikiza miyendo/ wongolani miyendo. tsamiritsani msana wanu ku chinthu.
- Dinani batani ili ngati mukufuna kuzimitsa makinawa. Dziwani kuti mukatero simukuyenera kuwagwiritsanso ntchito. Ngati mukufuna kufunsa kwa a dokotala musanazimitse makinawa imbani pama nambala awa;

Chenjezo.

- Makinawa akuyenera kuvalidwa mmwamba mwamkono ndipo onetsetsani kuti kachubu ka mpweya kapena chogwirizira mkono wanu chisazungulire pa khosi panu. Choncho ka chubu ka mphweya kakuyenera kuvalidwa kunja kwa chovala (ngakhale usiku umene).
- Samalirani kuti muonetsetse kuti ka chingwe ka chubu kasafinyike makamaka pamene mukugona.
- Makinawa asakhudzane ndi madzi. Ngati madzi angalowe mkati, makinawa sangathe kugwiranso ntchito, zikatero zimitsani makinawa ndikuchotsa mabatile.
- Kuwonongeka kwa makinawa kumadziwika pamene makinawa akulira mosalekeza. Ngati makinawa akulira mosalekeza, azimitseni potsatira malangizo ali mmwambamu, mkuwavula ndikudziwitsa adokotala mwansanga.
- Maliridwe ndi kagwiridwe tchito kamakinawa kakuyenera kufanana ndi mmene zinaliri pamene mumaphunzitsidwa. Ngati izi sizili choncho ndekuti pakhoza kukhala vuto pakalumikizidwe ka makina ndi kachubu zimene zingapangitse kukhala ndi zotsatila zolakwika.
